# Matrix Metalloproteinase-9 (MMP-9) in Psoriasis: Integrating Extracellular Matrix Remodeling, Neutrophil–Endothelial Crosstalk and Biomarker Evidence

**DOI:** 10.3390/medsci14040481

**Published:** 2026-08-14

**Authors:** Marta Wolosowicz, Slawomir Prokopiuk, Julia Nowowiejska-Purpurowicz, Tomasz W. Kaminski

**Affiliations:** 1 Thrombosis and Hemostasis Program, VERSITI Blood Research Institute, Milwaukee, WI 53226, USA; 2 Faculty of Science, University of Lomza, 14 Akademicka St., 18-400 Lomza, Poland; 3 Department of Dermatology and Venereology, Medical University of Bialystok, 15-540 Bialystok, Poland

**Keywords:** psoriasis, matrix metalloproteinase-9, extracellular matrix remodeling, neutrophils, vascular dysfunction, biomarker

## Abstract

Psoriasis is a chronic immune-mediated inflammatory disease in which cytokine-driven epidermal activation is accompanied by extensive remodeling of the extracellular matrix, basement membrane, and cutaneous microvasculature. Matrix metalloproteinase-9 (MMP-9; gelatinase B) may provide an important link between these structural alterations and several key features of psoriatic inflammation, including neutrophil activation, endothelial dysfunction, vascular hyperpermeability, and leukocyte recruitment. This review critically examines the biological and clinical relevance of MMP-9 across the spectrum of psoriatic disease. Particular focus is given to the cellular sources and regulation of MMP-9, its expression in lesional and non-lesional skin, and its interactions with the IL-23/Th17, IL-17, TNF-α, IL-36, MAPK, and NF-κB signaling pathways. Experimental studies support a functional neutrophil–MMP-9–endothelium axis in which MMP-9 can promote endothelial activation, vasodilation, vascular hyperpermeability, and leukocyte transmigration. However, this mechanistic evidence derives predominantly from experimental systems, whereas human studies mainly demonstrate associations between psoriasis and increased tissue or circulating MMP-9. The interpretation of circulating MMP-9 is further complicated by sample type, enzymatic activation state, assay heterogeneity, treatment exposure, and inflammatory and cardiometabolic confounders. Overall, MMP-9 represents a biologically possible contributor to psoriatic tissue remodeling and vascular inflammation, but its causal importance in human psoriasis remains insufficiently established. Future studies should determine whether MMP-9 can contribute to validated phenotype-specific multimarker models. At present, its clinical utility as either a biomarker or therapeutic target remains exploratory.

## 1. Introduction: Why MMP-9 Deserves a Focused Review in Psoriasis

### 1.1. Psoriasis Beyond Cytokines

Psoriasis is a chronic, relapsing, immune-mediated papulosquamous disorder that develops in genetically susceptible individuals under the influence of environmental and immunological triggers [1]. Its pathogenesis is driven by persistent activation of the dendritic cell–T cell–keratinocyte axis, which leads to epidermal hyperplasia, parakeratosis, and the formation of well-demarcated erythematous plaques covered by silvery scale [2,3]. Psoriasis is also one of the most prevalent chronic inflammatory skin diseases, although its distribution varies considerably across geographic regions. Recent population-based studies estimate an age-standardized global prevalence of approximately 0.8–1.0%, with the highest rates reported in North America, Western Europe, and Australasia, and substantially lower prevalence in several Asian and Western Pacific populations [4,5].

At the molecular level, psoriasis is described as a prototypical cytokine-driven dermatosis. The IL-23/Th17/IL-17 pathway, together with TNF-α, forms the central inflammatory network responsible for the initiation and persistence of psoriatic inflammation [2,6]. Environmental and tissue-derived danger signals, including damage-associated molecular patterns (DAMPs) and pathogen-associated molecular patterns (PAMPs), stimulate keratinocytes to release antimicrobial peptides (AMPs), which can activate dendritic cells (DCs) [2,6]. Activated DCs produce several cytokines, among which IL-23 is particularly important. IL-23 supports the maintenance and expansion of pathogenic Th17-type responses, while IL-17A and related effector cytokines act directly on keratinocytes. This signaling promotes excessive proliferation, abnormal differentiation, and the expression of chemokines, antimicrobial peptides, and additional inflammatory mediators. TNF-α reinforces these effects by enhancing IL-17-dependent responses in keratinocytes. The resulting bidirectional interaction between immune cells and the epidermis establishes a self-sustaining inflammatory loop that contributes to both the chronic course of psoriasis and the characteristic architecture of psoriatic plaques [7,8].

### 1.2. MMP-9 as a Missing Link Between Inflammation and Tissue Remodeling

Within this pathogenic landscape, matrix metalloproteinase-9 (MMP-9; gelatinase B) should not be recognized solely as an enzyme responsible for extracellular matrix degradation [9,10]. Rather, it functions as a pericellular protease at the intersection of tissue remodeling and inflammatory-cell trafficking [11]. Through cleavage of basement-membrane and interstitial matrix components, as well as selected cytokines, chemokines, growth factors, and adhesion-related molecules, MMP-9 may promote leukocyte migration while compromising epithelial and endothelial barrier integrity. These activities provide a mechanistic basis for linking matrix turnover with vascular activation, angiogenic signaling, and the structural consequences of persistent inflammation [12,13].

This perspective is particularly relevant in psoriasis, which involves extensive remodeling of epidermal, dermal, and vascular compartments. Human studies demonstrate increased MMP-9 expression in psoriatic lesions, whereas experimental studies provide functional evidence linking MMP-9 to extracellular-matrix remodeling and vascular dysfunction [14,15]. These findings support MMP-9 as a biologically plausible contributor to the psoriatic tissue environment [16]. However, current human data are predominantly observational and do not establish that MMP-9 independently drives plaque formation or persistence. MMP-9 should therefore be considered a candidate mediator of inflammatory remodeling whose relative contribution to human psoriasis remains to be defined.

### 1.3. Rationale and Scope of the Review

Previous reviews have examined matrix metalloproteinases across inflammatory skin disorders, while our recent review addressed MMP-9 from a broad pharmacological perspective across multiple disease settings, with particular emphasis on its regulation, direct and indirect inhibition, and therapeutic modulation by established or repurposed agents [11]. The present review differs substantially in both scope and purpose. Rather than reassessing MMP-9 as a general therapeutic target, it focuses specifically on psoriasis and integrates recent evidence concerning MMP-9 expression in lesional and clinically non-lesional skin, neutrophil–endothelial interactions, extracellular-matrix and vascular remodeling, and circulating biomarker measurements.

Importantly, the present review also examines MMP-9 across distinct psoriatic phenotypes, including plaque psoriasis, generalized pustular psoriasis, and psoriatic arthritis, and explicitly distinguishes evidence derived from patients with psoriasis from findings obtained in psoriasiform models or other experimental settings. These data are integrated into a psoriasis-specific conceptual framework linking cytokine-driven inflammation, neutrophil-derived MMP-9, endothelial dysfunction, extracellular-matrix remodeling, and biomarker potential. Sections addressing MMP-9 structure, activation, analytical limitations, and therapeutic inhibition are included only to provide the biological and translational context necessary for interpreting this psoriasis-specific evidence.

Throughout the review, we distinguish three levels of evidence: direct observations in patients with psoriasis or patient-derived material, experimental findings from in vitro or psoriasiform models, and general mechanistic observations derived from non-psoriatic biological settings. Findings from the latter two categories are not presented as direct evidence of a mechanism operating in patients with psoriasis.

**Figure 1:**
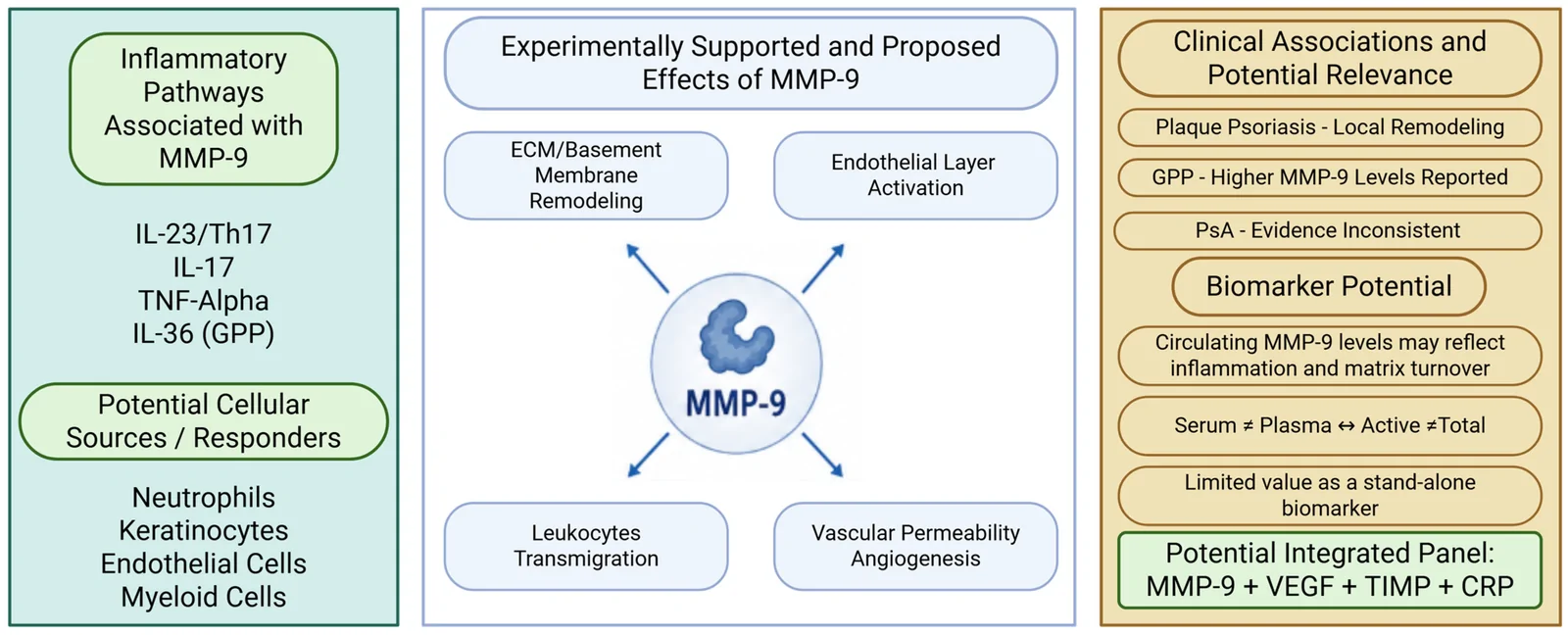
Proposed framework for MMP-9 in psoriasis. Cytokine-driven inflammation is associated with increased MMP-9 expression in psoriatic tissue and circulation. Experimental studies, particularly those using patient-derived neutrophils, endothelial-cell assays, and psoriasiform models, provide functional evidence linking MMP-9 to extracellular-matrix remodeling, endothelial activation, vascular permeability, and leukocyte transmigration. In contrast, human tissue and circulating biomarker studies are predominantly observational and do not establish a causal role for MMP-9 in psoriasis. Circulating MMP-9 should additionally be interpreted in the context of treatment exposure, analytical variability, systemic inflammation, and cardiometabolic confounding [17,18]. The figure therefore represents a proposed mechanistic framework rather than a fully validated pathway in human psoriasis. Abbreviations: CRP, C-reactive protein; ECM, extracellular matrix; GPP, generalized pustular psoriasis; PsA, psoriatic arthritis; TIMP, tissue inhibitor of metalloproteinases. *Biorender license: UV2A22R92A*.

A central objective is to distinguish observations made directly in patients with psoriasis from results obtained in psoriasiform animal models or extrapolated from the general biology of MMP-9. The review also examines several factors that may account for inconsistencies between studies, including the biological material analyzed, the activation state of the enzyme, binding to endogenous inhibitors, cellular source, prior treatment, and cardiometabolic comorbidities. By applying this more focused and critical framework, we seek to clarify which aspects of MMP-9 biology in psoriasis are supported by direct evidence and which remain plausible but insufficiently demonstrated. The proposed conceptual framework is summarized in Figure 1.

### 1.4. Literature Search Strategy

A narrative literature search was performed in PubMed/MEDLINE, Scopus, and Web of Science. The core search combined terms related to psoriasis and MMP-9, including (“psoriasis” OR “psoriatic arthritis” OR “generalized pustular psoriasis”) AND (“matrix metalloproteinase-9” OR “MMP-9” OR “MMP9”). Additional targeted searches combined these terms with “extracellular matrix”, “basement membrane”, “neutrophil”, “endothelial”, “vascular permeability”, “angiogenesis”, “IL-17”, “IL-23”, “TNF”, “IL-36”, “MAPK”, “NF-kB”, “biomarker”, “serum”, “plasma”, and “TIMP”. Search syntax was adapted to the requirements of each database. The final literature search was performed on 3 August 2026.

Priority was given to original studies published from 2021 to 2026 that directly evaluated MMP-9 in patients with psoriasis, patient-derived samples, or established psoriasiform experimental models. Earlier studies were retained when they provided important mechanistic, methodological, or historical information not adequately addressed by more recent publications. Eligible publications included original human studies, experimental studies, and selected reviews relevant to MMP-9 biology, psoriasis pathogenesis, analytical methodology, or therapeutic modulation.

Publications were excluded when they did not provide information relevant to MMP-9 or psoriatic disease, duplicated data already represented by another report, were available only as abstracts without sufficient methodological information, or were unrelated to the biological or analytical questions addressed in this review. Studies from non-psoriatic disease settings were included only when they provided necessary general mechanistic or methodological context and were not interpreted as direct evidence for psoriasis-specific mechanisms.

Titles and abstracts were screened for relevance, followed by full-text assessment of potentially eligible publications. Reference lists of the selected articles were also examined to identify additional relevant studies. Because this work was designed as a narrative rather than systematic review, no predefined systematic-review protocol, formal risk-of-bias assessment, or quantitative evidence synthesis was undertaken. Following reference-level classification, the included literature comprised approximately 17 primary studies based on patients with psoriasis or patient-derived material, 7 studies using psoriasiform experimental models, and 8 primary mechanistic or methodological studies from other biological settings; the remaining references consisted primarily of reviews and background literature. Studies combining human material and experimental models were classified according to their principal source of evidence.

## 2. Basic Biology of MMP-9 in Skin

### 2.1. Structure, Activation, and Inhibition

The general structure, activation, and pharmacological regulation of MMP-9 have been reviewed in detail previously [11] therefore, only features directly relevant to the interpretation of MMP-9 biology in psoriasis are summarized here. MMP-9 is produced mainly as an inactive precursor, pro-MMP-9. In this form, the N-terminal propeptide preserves enzymatic latency by blocking access to the zinc-containing catalytic site. Activation occurs when the propeptide is removed proteolytically or displaced through conformational change, allowing MMP-9 to cleave gelatin, denatured collagens, basement-membrane components, and several non-matrix substrates [19]. In addition to its catalytic domain, MMP-9 contains a C-terminal hemopexin-like domain that influences substrate recognition, interactions with other macromolecules, cellular localization, and the context in which proteolysis occurs.

MMP-9 activity is tightly controlled by tissue inhibitors of metalloproteinases. TIMP-1 is the major endogenous binding partner of pro-MMP-9 and inhibits the active enzyme, whereas TIMP-2 can suppress active MMP-9 but is more closely associated with MMP-2 regulation than with stable pro-MMP-9 complex formation [20,21]. This distinction is important because the amount of MMP-9 detected in a biological sample does not necessarily reflect its proteolytic activity. Measurements of total protein may include latent pro-MMP-9, active MMP-9, inhibitor-bound forms, and larger molecular complexes. Actual enzymatic activity depends on several additional factors, including zymogen activation, the local balance of inhibitors, oxidative modifications, and access to susceptible substrates [22].

### 2.2. Cellular Sources of MMP-9: Established and Putative Contributors

Among the cellular sources identified in psoriasis, infiltrating neutrophils and other myeloid cells currently have the strongest support as major contributors to MMP-9 within active lesions. Keratinocytes may also participate in its production, as suggested by epidermal immunostaining and earlier in vitro studies, although tissue-level staining cannot by itself establish definitive cellular origin [15]. Endothelial cells, fibroblasts, lymphocytes, and other resident or recruited cell populations are also plausible sources based on the broader biology of MMP-9 in inflammatory and cutaneous tissues. However, their relative contribution in human psoriasis has not yet been quantified with sufficient precision. The dominant source of MMP-9 is unlikely to be uniform across all lesions. It may instead depend on disease stage, clinical phenotype, prior or ongoing treatment, and the extent of neutrophil accumulation within the tissue [14,15].

### 2.3. Biological Functions Relevant to Psoriasis

Based on the general proteolytic properties of MMP-9, degradation of extracellular-matrix and basement-membrane components represents a plausible mechanism through which increased MMP-9 could influence psoriatic tissue remodeling. Direct demonstration of specific MMP-9-mediated substrate cleavage in human psoriatic lesions remains limited [11,12].

## 3. MMP-9 Expression in Psoriatic Skin

### 3.1. Lesional Skin: MMP-9 as a Marker of Active Plaque Remodeling

Support for this mechanistic role also comes from studies examining MMP-9 directly within psoriatic lesions. A recent histological and molecular analysis showed higher MMP-9 protein levels in plaque tissue than in both non-lesional skin from the same patients and skin from healthy controls without dermatoses. Immunostaining was particularly prominent within the epidermis [15]. These observations are relevant because they demonstrate that MMP-9 is present within the pathological tissue environment of the plaque, rather than being detectable only in the circulation or inferred from general inflammatory mechanisms.

Other studies have similarly reported increased MMP-9 expression at the transcript and protein levels in lesional psoriatic skin, supporting its involvement in the ongoing remodeling of the extracellular matrix. In parallel, experimental evidence that neutrophil-derived MMP-9 can activate cutaneous endothelial cells and increase vascular permeability provides a plausible link between local enzyme release and the inflammatory and vascular changes that characterize active psoriasis [14].

### 3.2. Non-Lesional Skin: Evidence for Altered MMP-9 Expression in Clinically Uninvolved Tissue

A cross-sectional tissue study found that MMP-9 protein expression was higher in clinically non-lesional skin from patients with psoriasis than in skin obtained from healthy controls without dermatoses [15]. This result is consistent with the view that apparently unaffected skin of patients with psoriasis may already display molecular abnormalities. It does not, however, provide direct evidence of a pre-lesional state. The control samples were derived from blepharoplasty tissue and were therefore not anatomically matched to the patient specimens. In addition, immunohistochemistry assessed protein abundance rather than gelatinolytic activity, and the sampled non-lesional sites were not followed prospectively to determine whether plaques subsequently developed in these regions [15].

Psoriatic skin contains tissue-resident memory T (TRM) cells, non-circulating lymphocytes that persist locally and may contribute to the recurrence of plaques at previously affected sites [23,24]. TRM-derived cytokines may stimulate MMP-9 production by resident or recruited cells. Conversely, MMP-9-mediated extracellular matrix remodeling could facilitate T-cell migration into peripheral tissues. These relationships remain mechanistically plausible but have not been established prospectively in clinically non-lesional skin. Based on the available data, however, it cannot be concluded that MMP-9 defines a pre-lesional field or predicts where future plaques will emerge [25,26].

### 3.3. Relationship to Histologic Hallmarks of Psoriasis

Increased MMP-9 expression should be interpreted with the insights into the characteristic histopathology of psoriasis, including acanthosis, parakeratosis, rete-ridge elongation, vascular remodeling, and inflammatory-cell infiltration [27]. Current evidence is consistent with a role for MMP-9 in matrix remodeling and inflammatory-cell trafficking, but does not establish it as a primary driver of epidermal hyperplasia or plaque architecture [27,28].

## 4. MMP-9, Neutrophils, and Vascular Activation in Psoriasis

These observations place MMP-9 within the remodeled tissue environment of psoriatic lesions, but they do not fully explain the inflammatory pathway through which the enzyme may exert its most immediate effects. Neutrophils are particularly relevant in this area because they constitute a rapidly available source of MMP-9 and are closely involved in leukocyte recruitment, endothelial activation, increased vascular permeability, and amplification of local inflammation. Their ability to release MMP-9 at sites of active tissue injury therefore positions them at an important functional interface between vascular responses and the evolving inflammatory architecture of the psoriatic plaque.

### Neutrophil-Derived MMP-9 and Endothelial Dysfunction

The principal mechanistic evidence for the neutrophil–MMP-9–endothelium axis comes from Chen et al. [14], a study combining patient-derived neutrophils, endothelial-cell experiments, and psoriasiform mouse models. In the human component, neutrophils from patients with psoriasis showed increased MMP-9 content and release. The causal effects of MMP-9 on endothelial ERK1/2 and p38 signaling, vascular permeability, T-cell transmigration, and the response to pharmacological MMP-9 inhibition were demonstrated primarily in experimental systems rather than directly in patients. Accordingly, the complete neutrophil–MMP-9–endothelium pathway should be regarded as a strongly supported experimental model that has not yet been fully demonstrated in human psoriatic lesions. Pharmacological inhibition of MMP-9 attenuated vascular abnormalities and inflammation in both imiquimod- and IL-23-induced models of psoriasiform dermatitis [14]. These experiments therefore provide the strongest direct support for a functional neutrophil–MMP-9–endothelium axis in psoriasis.

Related evidence supports the broader involvement of MMP-9-producing neutrophils in psoriatic inflammation. Subsequent work identified a CXCR4^high^ neutrophil population that accumulates in the circulation and inflamed skin of patients with psoriasis, correlates with disease severity, and displays enhanced degranulation and MMP-9 release [29]. These findings strengthen the concept that activated neutrophil subsets may represent an important source of MMP-9 in psoriasis, but they do not independently reproduce the complete endothelial mechanism described by Chen et al.

Accordingly, the proposed pathway linking neutrophil-derived MMP-9 with endothelial ERK1/2 and p38 activation, vasodilation, increased vascular permeability, and enhanced leukocyte transmigration should currently be regarded as a compelling but predominantly single-study mechanistic model [14]. To our knowledge, the complete pathway has not yet been independently replicated in another psoriasis cohort or experimental study. Independent validation, particularly in human lesional tissue and additional experimental models, will be necessary to determine how broadly this mechanism operates across psoriasis phenotypes and disease stages.

## 5. MMP-9 Within Cytokine and Signaling Networks

Beyond its role in matrix remodeling and vascular inflammation, MMP-9 must also be considered within the signaling networks that drive psoriasis. Its expression and activation are not independent events but are regulated by the same inflammatory pathways that sustain the disease. In particular, IL-17, IL-23, TNF-α, MAPK, and NF-κB signaling may influence both the production of MMP-9 and the biological effects that follow its release [14,28,29,30,31]. MMP-9 is therefore best viewed as a downstream proteolytic effector integrated into the broader cytokine circuitry of psoriatic inflammation.

### 5.1. Integration with TNF-α, IL-17, and IL-23 Pathways

MMP-9 can be viewed as one of the proteolytic effectors downstream of the cytokine networks that drive psoriatic inflammation. IL-17 and TNF-α induce a broad inflammatory response in keratinocytes and myeloid cells, including the expression of chemokines, antimicrobial peptides, adhesion molecules, and mediators involved in tissue remodeling. Together, these responses account for many of the characteristic transcriptional changes observed in psoriasis [6,30]. MMP-9 may contribute to the translation of these cytokine signals into structural and functional changes within the tissue by promoting matrix degradation, altering basement-membrane integrity, activating endothelial cells, and facilitating leukocyte migration.

In patient-derived monocytes, IL-17 and TNF-α were shown to regulate metalloproteinase production, with IL-17 increasing MMP-9 production and shifting the MMP-9/TIMP-1 balance toward greater proteolytic potential [31,32]. MMP-9 should therefore be considered an integral component of the IL-23/Th17/IL-17/TNF inflammatory network rather than an independent pathway or a passive end product of inflammation. Its induction provides one mechanism through which cytokine-driven immune activation can influence tissue remodeling and inflammatory-cell trafficking [31,32].

### 5.2. Correlation with Inflammatory Cytokines in Patients

In a clinical serum study, Michalak-Stoma et al. reported increased circulating MMP-9 concentrations in patients with psoriasis and associations with selected inflammatory mediators [33]. These observations demonstrate systemic association but do not establish the cellular source or enzymatic activity of MMP-9. They nevertheless indicate that altered MMP-9 concentrations are not confined to the skin and can be detected systemically alongside the inflammatory changes characteristic of psoriasis. Circulating MMP-9 may therefore reflect a broader inflammatory and tissue-remodeling profile rather than local epidermal or dermal proteolysis alone.

Taken together, these clinical data indicate that circulating MMP-9 accompanies the systemic inflammatory milieu of psoriasis, but they do not establish its cellular source, enzymatic activity, or a reciprocal causal effect of MMP-9 on cytokine signaling. The available psoriasis-specific evidence therefore supports cytokine-dependent regulation of MMP-9 production, particularly through IL-17- and TNF-associated pathways [32]. Whether MMP-9 reciprocally modifies these cytokine networks in human psoriasis remains insufficiently established.

### 5.3. MMP-9 as a Co-Regulated Component of MAPK/NF-κB-Associated Inflammation

Preclinical studies further suggest that MMP-9 operates within the inflammatory signaling networks of psoriasis rather than as an independent proteolytic pathway. In the imiquimod model, which reproduces several IL-23/IL-17-dependent features of psoriasiform inflammation, changes in MMP-9 are frequently observed alongside activation of MAPK- and NF-κB-related pathways [34]. This pattern indicates that extracellular matrix remodeling occurs in close association with canonical inflammatory signaling.

For example, biochanin A alleviated imiquimod-induced psoriasiform dermatitis while reducing MMP-9, IL-17A, and IL-23 levels and suppressing NF-κB and MAPK signaling [35]. Similarly, unconjugated bilirubin and a bilirubin derivative attenuated psoriasiform skin inflammation by decreasing neutrophil-associated MMP-9 activity, MAPK phosphorylation, pro-inflammatory cytokine expression, and neutrophil-directed chemokine responses [36]. These findings are consistent with an integrated cytokine-MAPK/NF-κB-protease network through which inflammation may promote leukocyte recruitment, tissue injury, and matrix remodeling [35,36]. However, because these compounds exert broad anti-inflammatory effects, the parallel reduction in MMP-9, cytokine production, and intracellular signaling demonstrates co-modulation rather than a direct causal sequence. The available data therefore does not establish MMP-9 inhibition as the principal mechanism responsible for the observed clinical or histological improvement.

## 6. Circulating MMP-9 as a Biomarker in Psoriasis

The role of MMP-9 in psoriasis may also extend beyond local tissue remodeling to its potential use as a circulating biomarker. Measurements in serum or plasma are of particular interest because they may reflect, although only indirectly, the combined effects of lesional inflammation, leukocyte activation, vascular dysfunction, and systemic inflammatory burden [33,37,38]. Circulating MMP-9 should therefore be considered not as a direct surrogate of cutaneous enzyme activity, but as a possible systemic readout of several processes contributing to psoriatic disease.

### 6.1. Cross-Sectional and Diagnostic Biomarker Evidence

Several cross-sectional studies have reported altered circulating MMP-9 in patients with psoriasis; however, demonstrating a difference between patients and controls is not equivalent to validating a diagnostic biomarker. Michalak-Stoma et al. reported higher serum MMP-9 concentrations in patients with psoriasis than in healthy controls [33], whereas Chihaoui et al. evaluated plasma MMP-9 together with VEGF, TIMP-2, and inflammatory variables in diagnostic models [37]. These findings provide evidence of disease association and exploratory diagnostic discrimination, respectively, but neither establishes MMP-9 as a clinically validated stand-alone diagnostic marker.

Studies of circulating MMP-9 generally indicate that psoriasis is accompanied by systemic dysregulation of metalloproteinases. Michalak-Stoma et al. reported significantly higher serum MMP-9 concentrations in patients with psoriasis than in healthy controls and examined their relationships with selected circulating cytokines and other MMPs [33]. These findings placed MMP-9 within a broader inflammatory profile rather than identifying it solely as a product of local matrix degradation.

More recently, Chihaoui et al. assessed VEGF, MMP-9, and TIMP-2 in 115 patients with psoriasis and 51 controls. Their results showed an association between circulating MMP-9 and psoriasis and suggested that the enzyme may contribute to multimarker models combining vascular, proteolytic, and inflammatory parameters [37]. Collectively, these human observations suggest that serum or plasma MMP-9 may reflect several components of psoriatic disease, including cutaneous inflammation, leukocyte activation, vascular dysfunction, and extracellular matrix turnover [37,38].

The interpretation of circulating MMP-9 nevertheless requires considerable caution. Values obtained from serum and plasma are not directly interchangeable, and serum concentrations may be influenced by the release of MMP-9 from leukocytes or platelets during coagulation [39]. Moreover, most immunoassays measure total MMP-9 protein and do not distinguish reliably between latent, active, inhibitor-bound, and complex forms of the enzyme.

### 6.2. Disease-Activity and Treatment-Response Evidence

Evidence for MMP-9 as a disease-activity or treatment-response biomarker is substantially more limited. In one longitudinal treatment study, plasma MMP-9 concentrations decreased following narrowband UVB phototherapy [38]. This finding supports treatment-associated modulation of circulating MMP-9, but does not demonstrate predictive utility, because baseline MMP-9 was not established as a marker identifying patients more likely to respond to therapy. Similarly, the available cross-sectional associations between MMP-9 and clinical or inflammatory variables are insufficient to validate MMP-9 as a longitudinal marker of psoriasis activity.

From a diagnostic perspective, circulating MMP-9 is more likely to be useful as part of a composite biomarker panel than as a stand-alone marker of psoriasis. Available ROC analyses provide preliminary support for multimarker approaches, but these models have not undergone adequate external validation. Earlier analyses of circulating MMPs and TIMPs identified discriminatory potential for selected metalloproteinase-related variables, whereas more recent work examining VEGF, MMP-9, TIMP-2, and an inflammatory Z-score suggested that combined models performed better than individual analytes [40].

This approach is biologically justified because psoriasis reflects the interaction of several inflammatory and vascular processes rather than dysregulation of a single protease. Within a multi-marker panel, MMP-9 may provide information on proteolytic remodeling, VEGF on angiogenic activity, TIMP-2 on metalloproteinase regulation, and CRP or related inflammatory scores on systemic inflammatory burden [37,40]. The diagnostic utility of MMP-9 may therefore depend less on defining a universal concentration threshold and more on combining it with markers that capture complementary aspects of matrix turnover, angiogenesis, and inflammation. Such a strategy could also limit some of the uncertainty associated with MMP-9 measurement alone, including variation related to sample type, leukocyte degranulation, platelet release, inhibitor binding, and the inability of most assays to distinguish total protein from enzymatically active MMP-9.

### 6.3. Analytical Limitations and Current Clinical Utility

Despite its potential as a circulating biomarker, MMP-9 is not yet sufficiently standardized for routine clinical use in psoriasis. Most studies have involved relatively small cohorts and have differed substantially in patient phenotype, disease severity, treatment history, and comorbidity profile. These differences limit the reproducibility of reported associations and make it difficult to define reliable diagnostic thresholds.

An additional source of variability is the biological material used for analysis. Serum and plasma MMP-9 concentrations are not directly comparable, because clotting and sample processing can induce the release of MMP-9 from leukocytes and platelets, resulting in higher serum values. Assay selection also affects interpretation. ELISA-based methods generally measure total immunoreactive MMP-9, whereas zymography, activity assays, and proteomic techniques assess different molecular forms or functional aspects of the MMP-9 system [41,42]. This distinction is important because total MMP-9 may include latent pro-MMP-9, active enzyme, TIMP-bound forms, and larger molecular complexes. Their combined abundance does not necessarily correspond to net proteolytic activity within psoriatic tissue [41,42].

Circulating MMP-9 is also not specific to psoriasis. Its concentration may be influenced by systemic therapy, ultraviolet phototherapy, obesity, smoking, acute infection, cardiometabolic disease, and cardiovascular inflammation, several of which are common in patients with psoriasis [38,43,44,45,46]. Current findings should therefore be interpreted as evidence of a systemic inflammatory and tissue-remodeling signal rather than validation of a disease-specific diagnostic assay. Future studies will require harmonized protocols covering sample type, anticoagulant selection, processing time, assay platform, and the reporting of active versus total MMP-9. Treatment status and major inflammatory and cardiometabolic confounders should also be incorporated consistently into study design and statistical analysis [43,44,45,46].

Importantly, the available studies address different biomarker concepts that should not be considered interchangeable. No standardized threshold externally validated diagnostic model or prospectively validated longitudinal application is currently available. The clinical utility of circulating MMP-9 should therefore be considered exploratory.

## 7. MMP-9 Across Psoriasis Phenotypes and Psoriatic Disease

The potential relevance of MMP-9 in psoriasis extends beyond chronic plaque disease. Psoriasis encompasses a heterogeneous group of cutaneous phenotypes and systemic manifestations, each of which may differ in the extent of tissue remodeling, leukocyte recruitment, vascular activation, and extracellular matrix turnover. This raises an important question as to whether MMP-9 primarily reflects a common inflammatory process shared across psoriatic disease, varies according to clinical phenotype, or serves as a broader marker of inflammation involving not only the skin but also the joints and comorbidity-related tissues.

### 7.1. Plaque Psoriasis Versus Generalized Pustular Psoriasis

Plaque psoriasis and generalized pustular psoriasis (GPP) differ substantially in their clinical presentation, genetic background, and inflammatory organization. For consistency, the term “plaque psoriasis” is used throughout this review for the common chronic plaque phenotype, whereas “psoriasis vulgaris” is retained only when referring to studies that used this terminology for their comparator population [47,48,49]. In plaque psoriasis, MMP-9 appears to be involved mainly in extracellular matrix remodeling, endothelial activation, and leukocyte recruitment within an IL-23/Th17/TNF-driven inflammatory environment [14,15]. GPP, in contrast, is a rare and potentially life-threatening neutrophilic dermatosis characterized by IL-36-dependent autoinflammation, marked epidermal neutrophil accumulation, and widespread pustule formation.

These phenotypic differences are also accompanied by differences in MMP-9 expression. A recent tissue proteomic study comparing GPP with a comparator group described by the investigators as psoriasis vulgaris found significantly higher MMP-9 levels in GPP, a result subsequently confirmed by immunofluorescence and Western blotting [50]. These observations suggest that MMP-9 may be particularly prominent in the neutrophil-rich inflammatory environment of GPP, where it could contribute to tissue proteolysis, vascular leakage, further leukocyte recruitment, and pustule development [50]. The evidence remains preliminary, however, and currently supports a phenotype-associated increase rather than establishing MMP-9 as a defining pathogenic driver of GPP. MMP-9 may therefore represent a shared molecular feature of plaque and pustular psoriasis, while its relative importance may differ across distinct inflammatory endotypes of psoriatic disease.

### 7.2. MMP-9 in Psoriatic Arthritis

Psoriatic arthritis (PsA) extends the disease process beyond the skin to the synovium, entheses, tendons, cartilage, and bone. These additional tissue compartments create a setting in which extracellular matrix turnover and protease activity may have particular pathogenic relevance. MMP-9 is a plausible contributor to inflammatory-cell migration, synovial invasion, angiogenesis, and osteochondral matrix degradation, although the clinical evidence in PsA is less consistent than that obtained from psoriatic skin [51].

Clinical studies of circulating MMP-9 in PsA have produced directly conflicting results. Prisila et al. evaluated 43 patients with PsA (14 active and 29 inactive) and 29 healthy controls and reported substantially higher circulating MMP-9 in both active (13.43 ± 3.99 ng/mL) and inactive PsA (12.56 ± 5.37 ng/mL) than in controls (5.71 ± 3.82 ng/mL) [51]. The corresponding mean differences versus controls were 7.77 ng/mL (95% CI 4.19–11.36) for active PsA and 6.91 ng/mL (95% CI 4.00–9.80) for inactive PsA. However, MMP-9 did not differ between active and inactive disease and did not correlate significantly with either CPDAI or DAPSA. The reported ROC analysis yielded an AUC of 0.88 (95% CI 0.796–0.964) for distinguishing PsA from healthy controls, but this estimate was derived and tested in the same cohort, without reported resampling-based internal validation or validation in an independent cohort. It should therefore be regarded as exploratory rather than as evidence of established diagnostic performance.

In contrast, Veale et al. examined a larger cohort of 97 patients with PsA and 15 healthy controls using a Meso Scale Discovery multiplex assay and found significantly lower serum MMP-9 in PsA than in controls (*p* < 0.01) [52]. The study did not report a numerical effect estimate or confidence interval for this PsA-versus-control MMP-9 difference. Disease activity was stratified using DAS28-CRP (>3.2 versus <3.2), and MMP-9 showed only a non-significant tendency toward higher concentrations in patients with greater disease activity (*p* = 0.07). Importantly, treatment exposure differed substantially between cohorts. In the Prisila study, 33 of 43 PsA patients received conventional DMARDs and only one received a biologic DMARD, whereas in the Veale cohort approximately 10% received biologic DMARDs, 11% conventional DMARDs, and approximately 76% were untreated; stratified analyses indicated that patients receiving biologics showed the greatest reduction in MMP-9. These differences in treatment exposure, disease-activity classification, analytical platform, and preanalytical handling may contribute to the opposing direction of the reported effects.

Sample handling represents an additional source of uncertainty. Veale et al. explicitly analyzed serum, whereas Prisila et al. referred to serum MMP-9 but described collection into sodium-heparin tubes followed by isolation of plasma. Given the known sensitivity of circulating MMP-9 measurements to biospecimen type and processing, this methodological inconsistency further limits direct comparison between the studies. Overall, the available evidence does not establish either increased or decreased circulating MMP-9 as a reproducible feature of PsA, and current diagnostic findings should be considered preliminary. Evidence based on extracellular matrix degradation products may be more informative. MMP-generated fragments, including MMP-cleaved prolargin, have been reported to be elevated in PsA and can be quantified as neo-epitope biomarkers, supporting the presence of active-matrix proteolysis in psoriatic joint disease [53]. At present, MMP-9 is therefore best regarded as a plausible participant in joint and entheseal remodeling and as a potential component of broader biomarker panels assessing extracellular matrix turnover. The available evidence does not yet support its use as a validated stand-alone marker of PsA diagnosis or disease activity.

### 7.3. Systemic Inflammation and Comorbidities

Evidence from non-psoriatic studies shows that circulating MMP-9 can be influenced by smoking and metabolic dysfunction [43,44]. Because cardiometabolic comorbidities are common in psoriasis [45,46], these factors represent important potential confounders in psoriasis biomarker studies. In patients with psoriasis and cardiometabolic comorbidities, MMP-9 should therefore be considered as a nonspecific but biologically plausible indicator of systemic matrix turnover and low-grade vascular inflammation. Current evidence does not support interpreting elevated MMP-9 as proof of a distinct cardiovascular or thrombotic mechanism attributable specifically to psoriasis.

## 8. Experimental Models and Therapeutic Modulation of MMP-9

Experimental models provide a useful bridge between descriptive observations in human tissue and conclusions regarding therapeutic relevance. They allow inflammatory triggers, cellular contributors, and treatment effects to be studied under more controlled conditions. In psoriasiform inflammation, changes in MMP-9 should therefore not be interpreted solely as evidence of direct enzyme inhibition. More often, they occur as part of a broader modification of the inflammatory response involving cytokine signaling, neutrophil activation, endothelial dysfunction, vascular permeability, and extracellular matrix remodeling.

### 8.1. IMQ-Induced Psoriasis-like Inflammation Murine Model

The imiquimod-induced mouse model is widely used to study psoriasis-like inflammation because it allows cytokine activation, epidermal hyperplasia, leukocyte infiltration, vascular changes, and matrix remodeling to be followed within a defined experimental timeframe. Although the model is acute and does not capture the chronic course or genetic complexity of human psoriasis, it reproduces several key features of psoriasiform pathology and depends substantially on IL-23/IL-17 signaling.

In this setting, the study by Noddeland et al. provides a useful overview of the metalloproteinase response to IMQ-induced inflammation [28]. Using targeted proteomics, the authors found increased levels of MMP-2, MMP-7, MMP-8, and MMP-13 in inflamed skin, whereas MMP-3, MMP-9, and MMP-10 were detected only after IMQ exposure. These findings indicate that psoriasiform inflammation induces a coordinated proteolytic response rather than an isolated change in a single enzyme. The appearance of MMP-9 exclusively in inflamed skin is particularly relevant, as it supports its classification as an inflammation-associated protease involved in active tissue remodeling rather than as a constitutively expressed structural enzyme [28].

The results should nevertheless be interpreted with caution. Targeted proteomics measures protein abundance and does not establish whether the detected MMP-9 was enzymatically active. Nor does its selective detection in inflamed tissue demonstrate that MMP-9 is independently responsible for the observed pathology. The IMQ model therefore strengthens the rationale for investigating MMP-9 in psoriasis, while also placing it within a broader metalloproteinase network that includes gelatinases, stromelysins, matrilysins, collagenases, and their endogenous inhibitors.

### 8.2. Direct and Indirect MMP-9 Inhibition in Preclinical Psoriasis

Therapeutic modulation of MMP-9 in experimental psoriasis can occur through two broad mechanisms: direct inhibition of its enzymatic activity or indirect suppression of its expression, release, and activation through upstream anti-inflammatory pathways. In the mechanistic study discussed in Section 4, pharmacological inhibition of MMP-9 reduced vascular permeability and attenuated inflammation in both IMQ- and IL-23-induced models [14]. These findings suggest that MMP-9 is not simply a by-product of tissue injury but can contribute functionally to vascular and inflammatory pathology. Direct therapeutic targeting nevertheless remains challenging. Earlier metalloproteinase inhibitors were limited by poor selectivity and the complex physiological functions of this enzyme family. Although more selective allosteric inhibitors of MMP-9 have shown promise in other inflammatory conditions, this approach has not yet been developed meaningfully for psoriasis [54].

Most preclinical studies in psoriasis have instead examined the indirect modulation of MMP-9 by compounds with broader anti-inflammatory effects. Biochanin A reduced IMQ-induced psoriasiform inflammation and decreased MMP-9, IL-17A, and IL-23 expression while suppressing NF-κB and MAPK signaling. Unconjugated bilirubin and its derivative similarly improved psoriasiform dermatitis while reducing neutrophil-associated MMP-9 activity and MAPK phosphorylation [35,36]. In these studies, changes in MMP-9 occurred alongside modulation of cytokine signaling, neutrophil activation, oxidative stress, and vascular responses, making it difficult to attribute therapeutic benefit specifically to MMP-9 suppression. Overall, the preclinical evidence identifies MMP-9 as a modifiable component of psoriasis-like inflammation. Further studies are needed to determine whether effective therapeutic intervention requires direct enzyme inhibition, upstream suppression of MMP-9-inducing pathways, or a combined strategy targeting both proteolysis and vascular inflammation.

### 8.3. Translational Caution: Why MMP-9 Is Not Yet a Clinical Psoriasis Target

**Figure 2:**
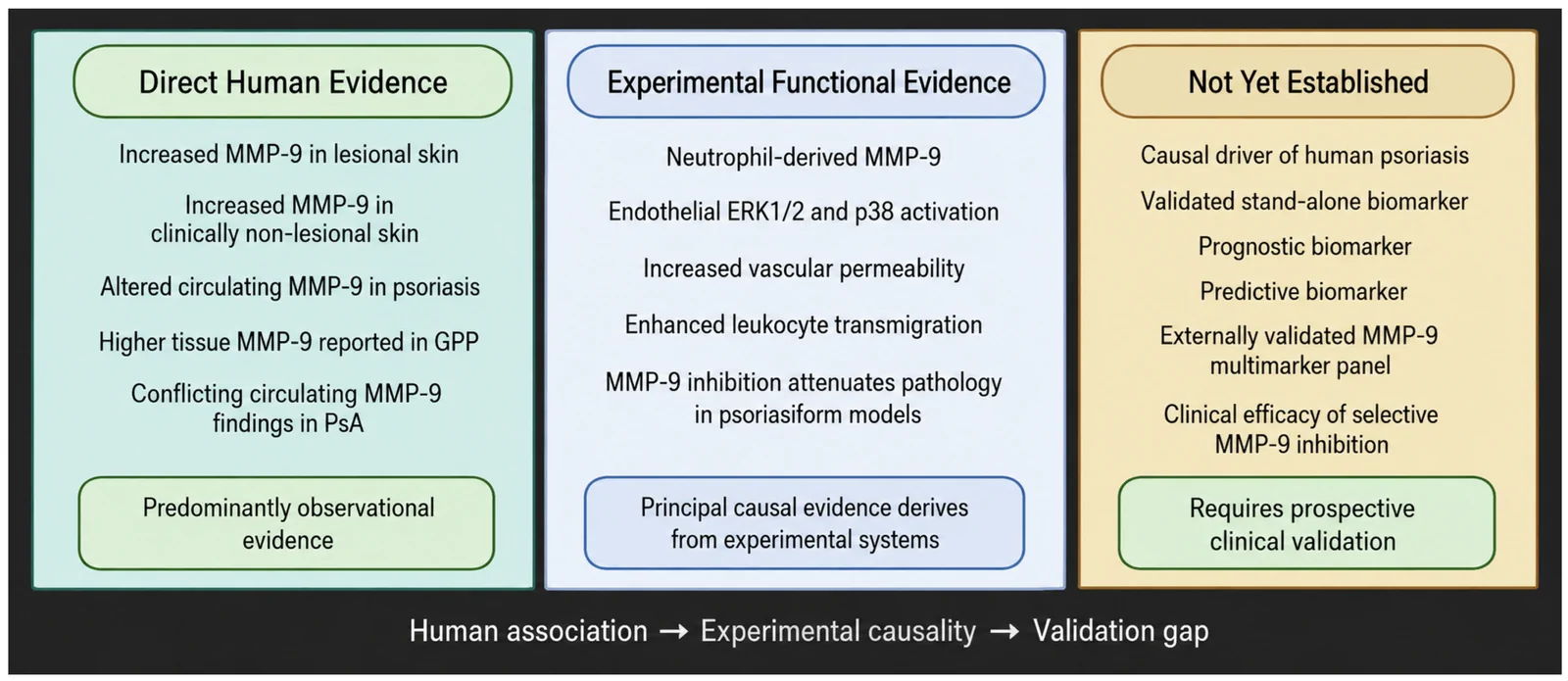
Current level of evidence supporting the biological and clinical relevance of MMP-9 in psoriatic disease. Human studies predominantly demonstrate associations between MMP-9 and psoriatic phenotypes, whereas the strongest functional evidence derives from experimental models. Major translational questions, including prognostic and predictive biomarker utility, external validation of multimarker models, and therapeutic efficacy of selective MMP-9 inhibition, remain unresolved. *Biorender license: QA2A23PMT5*.

The broader pharmacology and historical limitations of MMP-9 inhibition have been discussed in detail elsewhere [11]. In psoriasis, current evidence is limited to experimental models, in which pharmacological or indirect suppression of MMP-9 can attenuate selected inflammatory and vascular abnormalities. These findings do not establish MMP-9 as a therapeutic target in patients with psoriasis. Psoriasis-specific clinical evidence for selective MMP-9 inhibition is currently lacking, and studies of selective inhibitors in other inflammatory diseases demonstrate pharmacological feasibility rather than efficacy in psoriasis [54,55,56]. Future work should first determine whether particular psoriatic phenotypes show sufficient MMP-9 dependence to justify translational therapeutic studies as shown in Figure 2.

## 9. Future Directions and Conclusions

### 9.1. Standardization of MMP-9 Measurement

A major priority for future research is the standardization of MMP-9 measurement across the spectrum of psoriatic disease. MMP-9 should not be treated as a single analytical variable, because total immunoreactive protein, latent pro-MMP-9, active enzyme, inhibitor-bound forms, and proteolytic balance indices such as the MMP-9/TIMP-1 or MMP-9/TIMP-2 ratio provide different biological information [57]. This distinction is critical, as elevated MMP-9 protein in tissue or blood does not necessarily indicate increased enzymatic activity. It also does not reveal whether the measured signal originates from keratinocytes, infiltrating leukocytes, endothelial cells, or ex vivo release during sample collection and processing.

Future studies should therefore integrate tissue-based analyses with serum or plasma measurements and direct or surrogate assessments of gelatinolytic activity, preferably within the same well-characterized patient cohorts [58]. Preanalytical variables must also be reported consistently, including biospecimen type, anticoagulant, time to processing, storage conditions, and the number of freeze–thaw cycles. Equally important is a clear description of what each analytical platform measures, whether total protein abundance, zymographic activity, proteomic detection, or spatially resolved tissue expression. Greater methodological harmonization would make it possible to determine more reliably whether MMP-9 functions primarily as a pathogenic mediator, a biomarker candidate, or a treatment-responsive indicator in psoriasis.

### 9.2. MMP-9 as Part of a Multi-Marker Panel

At present, MMP-9 has no validated stand-alone or multimarker clinical application in psoriasis. Exploratory studies combining MMP-9 with vascular, metalloproteinase-related, or inflammatory variables suggest that composite approaches warrant further investigation [37,40]. However, no MMP-9-centered model has undergone adequate external validation, and differences in sample type, analytical methodology, treatment exposure, and clinical phenotype remain important barriers to translation. Future studies should therefore evaluate MMP-9 prospectively within standardized, longitudinal, and phenotype-specific biomarker models rather than assume near-term clinical utility.

### 9.3. Conclusions

Overall, current evidence positions MMP-9 as a link between inflammatory signaling, extracellular-matrix remodeling, neutrophil activity, and vascular dysfunction in psoriasis. The strongest causal evidence concerns neutrophil-derived MMP-9 and endothelial dysfunction in experimental psoriasis models, whereas human biomarker and phenotype-specific data remain heterogeneous. MMP-9 should therefore be regarded as a mechanistically relevant component of psoriatic inflammation and a candidate for multimarker approaches rather than as an established stand-alone biomarker or therapeutic target. These findings are summarized in Supplementary Table S1.

## Data Availability

No new data were created or analyzed in this study. Data sharing is not applicable to this article.

## Supplementary Materials

The following supporting information can be downloaded at https://www.mdpi.com/article/10.3390/medsci14040481/s1. Table S1: Summary of current evidence regarding the biological and clinical relevance of MMP-9 in psoriatic disease.

## Abbreviations

AMP	antimicrobial peptide
AUC	area under the curve
CD4+	cluster of differentiation 4-positive
CRP	C-reactive protein
CXCR4	C-X-C chemokine receptor type 4
DAMP	damage-associated molecular pattern
DC	dendritic cell
ECM	extracellular matrix
ELISA	enzyme-linked immunosorbent assay
ERK1/2	extracellular signal-regulated kinases 1 and 2
GPP	generalized pustular psoriasis
IL	interleukin
IL-17	interleukin-17
IL-17A	interleukin-17A
IL-23	interleukin-23
IL-36	interleukin-36
IMQ	imiquimod
MAPK	mitogen-activated protein kinase
MMP	matrix metalloproteinase
MMP-9	matrix metalloproteinase-9
NB-UVB	narrowband ultraviolet B
NF-κB	nuclear factor kappa-light-chain-enhancer of activated B cells
PAMP	pathogen-associated molecular pattern
PASI	Psoriasis Area and Severity Index
PsA	psoriatic arthritis
ROC	receiver operating characteristic
Th17	T helper 17
TIMP	tissue inhibitor of metalloproteinases
TIMP-1	tissue inhibitor of metalloproteinases-1
TIMP-2	tissue inhibitor of metalloproteinases-2
TNF-α	tumor necrosis factor alpha
TRM	tissue-resident memory T cell
UVB	ultraviolet B
VEGF	vascular endothelial growth factor
