# Supplementary material for: Matrix Metalloproteinase-9 (MMP-9) in Psoriasis: Integrating Extracellular Matrix Remodeling, Neutrophil–Endothelial Crosstalk and Biomarker Evidence
Source: Med Sci (Basel). 2026 Aug 14;14(4):481. doi: 10.3390/medsci14040481 (PMC13515184; doi:10.3390/medsci14040481)
Supplement: Supplementary file 1 [file medsci-14-00481-s001.zip › medsci-4466397-supplementary.pdf]

Table S1. Summary of current evidence regarding the biological and clinical relevance of MMP-9 in psoriatic disease

Evidence domain	Study setting / material	Main findings	Proposed significance of MMP-9	Key limitations	References
**Lesional skin expression**	Human psoriatic plaques compared with non-lesional and healthy skin	Increased MMP-9 expression was demonstrated in psoriatic lesional skin using tissue-based protein and gene-expression approaches.	Supports local involvement in extracellular-matrix remodeling, basement-membrane alteration, and plaque maintenance	Protein abundance does not necessarily indicate enzymatic activity; cell-specific origin is not always resolved	[15,23]
**Clinically non-lesional skin**	Human clinically uninvolved psoriatic skin	Higher MMP-9 expression than in control skin	May indicate disease-associated molecular alteration beyond visible plaques	Anatomically unmatched controls, cross-sectional design, and absence of longitudinal assessment prevent interpretation as a pre-lesional marker	[15]
**Neutrophil-endothelium axis**	Human neutrophils, endothelial-cell assays, and psoriasiform models	Neutrophil-derived MMP-9 activated endothelial cells, increased vasodilation and vascular permeability, and facilitated T-cell transmigration	Provides direct mechanistic support for MMP-9 as a vascular-inflammatory amplifier	The relative contribution of this pathway across human psoriasis phenotypes and disease stages remains uncertain	[14]
**Cytokine and signaling networks**	Human monocytes and experimental psoriasis-like inflammation	IL-17 and TNF-α increased metalloproteinase production; MMP-9 changes occurred alongside MAPK and NF-κB activation	Positions MMP-9 downstream of, and integrated within, canonical psoriatic inflammatory pathways	Most interventions were pleiotropic; co-modulation does not establish a direct causal hierarchy	[35,37,38]
**Circulating biomarker**	Serum and plasma studies in patients with psoriasis	Circulating MMP-9 was elevated in several cohorts and decreased following narrowband UVB therapy in one study	May reflect systemic inflammation, leukocyte activation, vascular perturbation, and matrix turnover	Serum and plasma are not interchangeable; assays often measure total rather than active MMP-9; substantial clinical and analytical heterogeneity	[50]
**Generalized pustular psoriasis**	Tissue proteomics, immunofluorescence, and Western blotting	MMP-9 expression was higher in GPP than in psoriasis vulgaris and decreased after IL-36 receptor inhibition	Suggests enrichment of MMP-9 in neutrophil-dominant and IL-36-associated inflammation	Evidence remains limited and does not establish MMP-9 as a defining driver of GPP	[51-53]
**Psoriatic arthritis**	Serum biomarker studies and ECM neo-epitope analyses	Studies reported inconsistent changes in circulating MMP-9, while MMP-generated matrix fragments supported active tissue proteolysis	MMP-9 may contribute to synovial, entheseal, and osteochondral remodeling and could complement ECM-turnover panels	Opposite effect directions, differences in treatment exposure, disease-activity definitions and assay methodology, and absence of external validation of diagnostic performance	[43-46]
**Cardiometabolic comorbidity**	Non-psoriatic cardiometabolic studies and psoriasis cohorts with cardiometabolic comorbidity	Circulating MMP-9 is influenced by smoking and metabolic dysfunction in non-psoriatic populations, while cardiometabolic comorbidities are common in psoriasis and may therefore confound circulating MMP-9 measurements	May contribute to a systemic inflammatory-remodeling signature in psoriasis with comorbid disease	Highly nonspecific and unable to distinguish psoriasis-related inflammation from concurrent cardiometabolic processes	[43-46]
**Experimental models and therapeutic modulation**	IMQ- and IL-23-induced psoriasiform inflammation	MMP-9 was induced in inflamed skin; direct inhibition reduced vascular and inflammatory pathology, while several anti-inflammatory compounds indirectly reduced MMP-9	Supports MMP-9 as a functionally modifiable component of psoriasis-like inflammation	Acute models do not reproduce the full complexity of human psoriasis; indirect suppression does not prove MMP-9-dependent efficacy	[14,28,32,33]
**Translational biomarker strategy**	Multianalyte and methodological studies	Combined models incorporating MMP-9, VEGF, TIMPs, inflammatory markers, or clinical variables appeared more informative than isolated measurements	Supports incorporation of MMP-9 into phenotype-informed multimarker panels	No MMP-9-centered panel has yet been externally validated or standardized	[37,40]

Table S1. Summary of current evidence regarding the biological and clinical relevance of MMP-9 in psoriatic disease. The table integrates findings from human tissue studies, circulating biomarker analyses, experimental models, and phenotype-specific investigations. Proposed biological and clinical implications are presented together with the principal methodological and translational limitations of the available evidence. References correspond to the numbering used in the main text.
